# Synchronous Control of a Group of Flying Robots Following a Leader UAV in an Unfamiliar Environment

**DOI:** 10.3390/s23020740

**Published:** 2023-01-09

**Authors:** Konrad Wojtowicz, Przemysław Wojciechowski

**Affiliations:** Faculty of Mechatronics, Armament, and Aerospace, Military University of Technology, 00-908 Warsaw, Poland

**Keywords:** drone, UAV, multi-agent, ArUco, markers, group of drones, machine vision

## Abstract

An increasing number of professional drone flights require situational awareness of aerial vehicles. Vehicles in a group of drones must be aware of their surroundings and the other group members. The amount of data to be exchanged and the total cost are skyrocketing. This paper presents an implementation and assessment of an organized drone group comprising a fully aware leader and much less expensive followers. The solution achieved a significant cost reduction by decreasing the number of sensors onboard followers and improving the organization and manageability of the group in the system. In this project, a group of quadrotor drones was evaluated. An automatically flying leader was followed by drones equipped with low-end cameras only. The followers were tasked with following ArUco markers mounted on a preceding drone. Several test tasks were designed and conducted. Finally, the presented system proved appropriate for slowly moving groups of drones.

## 1. Introduction

Due to global technological development and commercial opportunities, vast growth in the unmanned aerial vehicle market has been observed. Software, control systems, structures, and methods of analyzing the environment with drones are developing. There are high hopes for using drones for rescue and medical purposes. Thanks to the commercialization of the drone market, emergency services receive professional tools that make their work faster, easier, and safer. Often, after a fire, earthquake, or collapse, it is difficult or even impossible to assess the level of damage and determine the necessary action based on external observations. Using drones equipped with multispectral observation heads is recommended to monitor the situation inside buildings. Unmanned aerial vehicles, transmitting live, high-definition images, e.g., from a collapsed building or mining collapse, minimize the risk and do not expose rescuers to unnecessary danger while simultaneously offering first aid.

**Figure 1:**
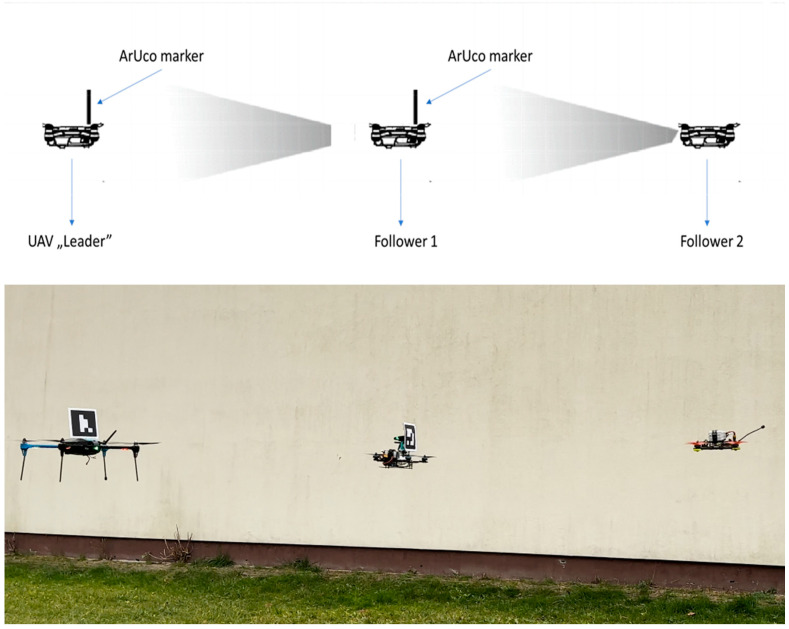
Assumptions of the designed system.

In rescue operations, particular attention is paid to the time needed to provide help. Drones searching for injured persons often cannot carry additional cargo in the form of essential materials such as bandages, medications, or even water. This paper presents a system of underequipped “follower” drones tracking a “leader” drone. The leader is equipped with systems enabling the identification and avoidance of obstacles and the location of the injured, in order to immediately provide the necessary means of survival for the victims of disasters (Figure 1). An example application of a group of drones is a mobile crop-monitoring system employing several drones in a group carrying optical sensors [1]. A comprehensive review listing other multi-agent system applications was presented in [2].

The project aimed to investigate whether it is possible to send several drones into unknown surroundings and control them via UAV “leader” tracking using ArUco tags. This seems to be the simplest and least expensive method, providing a considerable number of resources necessary for survival to people trapped in hard-to-reach or dangerous environments without risking the health or lives of rescuers. The main innovation is the structure of the multi-agent group of UAVs, wherein raising the number of inexpensive followers increases neither the data exchange between actors nor the computational power requirements.

## 2. Related Works

Regarding autonomous flights, the research can be divided into two areas. The first typically focuses on improving and increasing the efficiency of control algorithms. The second deals with teaching UAVs that have previously been operated in manual mode to “learn by heart”, recreating the required trajectory with corrections from external threat monitoring systems. Of course, there are many commercial structures wherein it is possible to use GPS. However, we are more interested in scenarios wherein the environment and surroundings through which one is to move are unknown, and it is impossible to estimate the position based on GNSS systems. Many works have indicated that solutions involving laser scanners, RGB-D sensors, or ultrasonic sensors mounted on the UAV board are fundamental and most effective. There are also solutions employing a synthetic aperture radar in addition to optical sensors [3].

It should be noted, however, that such solutions take up much space on the supporting structure of the drone, and their weight makes it impossible to take on additional cargo. Another approach is the simultaneous monitoring and remote control of each vehicle in the group [4]. Theoretically, it is possible to plan a trajectory for multiple drones in a constraint area [5]. However, in a natural environment, flights are only collision-free for a short time, because of unexpected disturbances. In the case we tested, i.e., small drones that can carry a small load, each gram of equipment is essential. According to many research results, the best solution is to track another UAV leader [6,7,8,9,10,11]. A multifunctional group of drones can be achieved by providing situational awareness by mounting sensors on the leader drone only [12] and a variety of mission sensors onboard group-member vehicles [13], e.g., to deliver enhanced scanning capabilities to infrastructure inspection systems [14] or build models of ancient sites [15].

A group of UAVs can be controlled by one of multiple formation strategies and techniques [16]; for rescue missions within disaster management systems, these include: virtual structure [17,18], consistency algorithm [19], behavior-based control [20], and leader–follower techniques. There are many advantages of using biological models, behavior-based formation control, and tracking, which give custom roles to particular agents [21,22,23]. However, these models can be adapted to swarms of vehicles and require constant agent-to-agent or agent-to-ground communication. In our case, we needed to bring a group of drones to a destination without putting additional tasks in their way. In the leader–follower formation, the followers can stay passive without any communication link. Various proposals exist for keeping the group together and moving forward in a leader–follower formation [24,25,26,27]. Many have introduced novel methods, algorithms, and ideas for controlling agents and the group. One of these, a linear consistency algorithm based on the leader–follower technique, was presented in [28]. It comprised a method of tracking the leader’s position, heading, and speed. In [29], a virtual piloting leader algorithm was designed. It successfully coped with a leader failure but required high computational power onboard all the agents. Further, a distributed UAV formation control method was designed in [30]. However, the applied higher-order consistency theory required a preorganized communication topology.

A summary of the simulations and applications of the leader–follower technique highlights a significant disadvantage: a high dependence on the leader. Any failure of the leader affects the mission. Another problem is the substantial computational power requirement when the number of agents rises. In this work, we addressed the second issue as this project’s main innovation, since the structure of the leader and followers does not change according to the number of agents in the group.

Visual passive markers are commonly used in every area of life. The visual pattern was first proposed in 1948 by two students, Bernard Silver and Norman Joseph Woodland. Due to the lack of appropriate technology, it was almost 30 years later that the barcode was used commercially and automatically.

**Figure 2:**
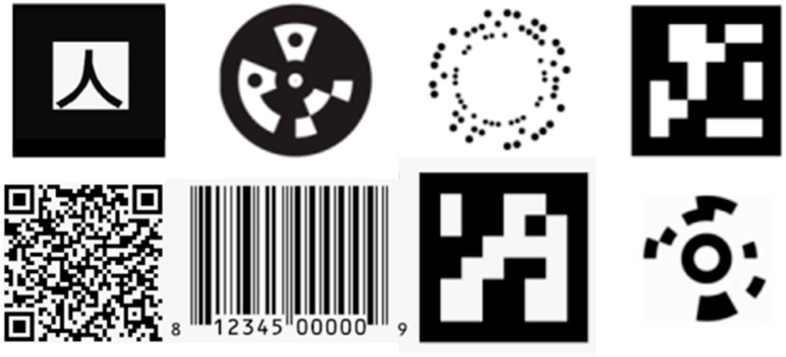
Eight existing marker systems. Top, from left to right: ARToolKit, Circular Data Matrix, RuneTag, ARToolKit Plus. Bottom, from left to right: QR code, barcode, ArUco, Cantag.

Currently, markers are commonly used to mark goods, position machines in production, or read the position and orientation of medical devices during minimally invasive treatment, primarily due to their low production cost (Figure 2).

One of the best-known passive markers is a QR code [31]. A large amount of information can be stored under one tag, making it suitable for data transfer. In addition, they are resistant to damage, which means that depending on the percentage of damage to the entire code, it is possible to read at least part of the data contained in it.

Another type of tag is one used to track objects. ARTag and Artoolkit are characterized by the speed of detection and easy tracking, but they are not immune to changes in lighting. The ArUco marker was designed with similar technology. Its advantage is that it generates a small percentage of false-positive recognition, while the method of its encoding increases the effective recognition distance and the number of bit conversions. Some works indicate that these are the most effective candidates for use in AR [32,33]. ArUco markers proved to be solid reference points for mobile test beds [34] and stationary test benches [35]. The markers provided a reliable reference for position and attitude determination, which could be enhanced by setting the markers in three-dimensional patterns [36]. The accurate positioning of the markers allows their application as characteristic points to support simultaneous localization and mapping systems [37]. We applied ArUco markers as reference points in our previous research on a drone automatic landing system [38].

Researchers from the University of Michigan built a square-shaped AprilTag with a barcode, similar to QR codes and ArUco. A significant problem with their use seems to be the low recognition speed. Olson et al. proposed an updated version of AprilTag called AprilTag2, which resulted in increased detection speed [39]. Another modification was a circular ring marker that was tested for efficiency but lacked feature recognition [40].

The CircularTag, WhyCon, RUNE-Tag, and TRIP tags present a different approach. These round tags allow for high positioning accuracy but involve a complex, system-intensive detection algorithm [41,42,43,44,45].

## 3. Materials and Automatic Control Algorithm

### 3.1. Test System Design

A classic X-shaped quadrotor was used for the tests. A Raspberry Pi 4 minicomputer was used as the system for the implementation of the detection and tracking program. Due to the desire to reduce costs, a HAMA C400 in Full HD 1080p webcam with a 70° visual angle was responsible for recording the image. The design was based on our previous research experience with drone airframes and flight controllers [46].

**Figure 3:**
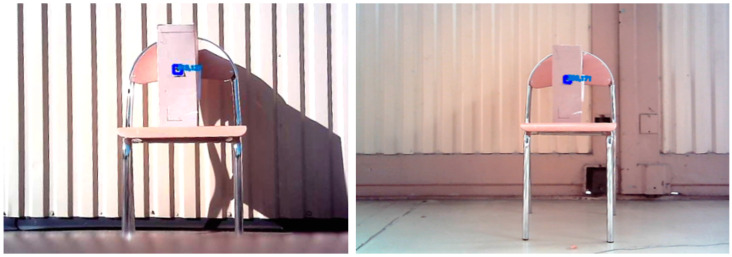
The marker identification rate versus distance.

**Figure 4:**
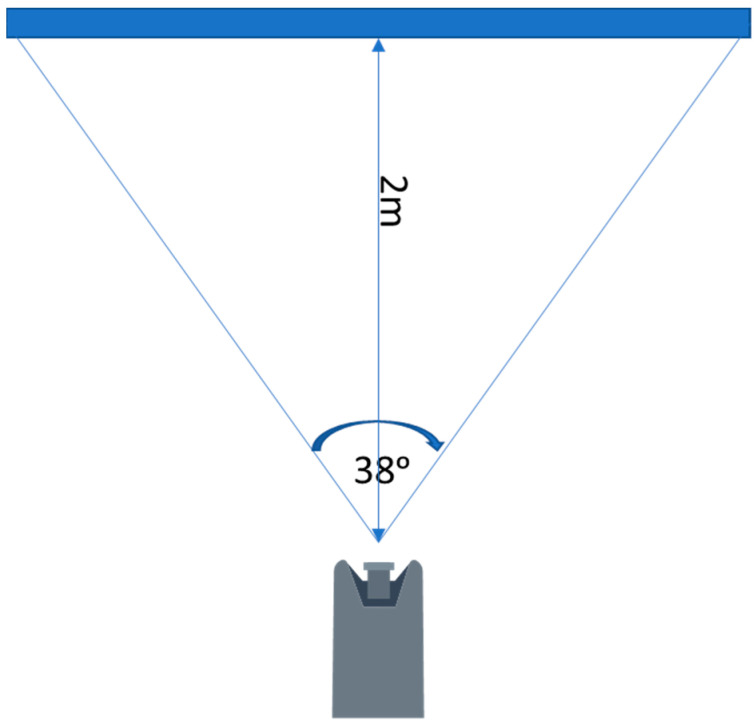
Effective camera field of view.

In the initial part of the study, a test of the limit values of the system was carried out (Figure 3). The main parameters that were determined during the tests were the maximum effective detection distance of the marker (Table 1 and Table 2), the maximum marker detection angle (change in marker position in the field of view of the camera), and the possibility of marker detection depending on its position in the camera’s field of view at the horizontal level. The marker detection was checked 2 m from the camera, in order to represent a system working in limited space. The effective detection angle of the markers at the horizontal level was 38ᵒ (Figure 4).

Three sizes of ArUco marker (3 cm × 3 cm, 6 cm × 6 cm, and 12 cm × 12 cm) were tested at different light intensities (770 lx and 63.7 klx).

Based on the obtained results, it was concluded that the attempts to track the markers in flight would be carried out only for markers with dimensions of 12 × 12 cm. This was due to the quick recognition of the marker and the ability to maintain tracking more easily at greater distances (maximum over 5.5 m) and with more pronounced changes in the leader’s course (52 left and 57 right in clear weather conditions), allowing the drones to follow the leader on more complicated routes.

### 3.2. Marker Detection Algorithm

**Figure 5:**
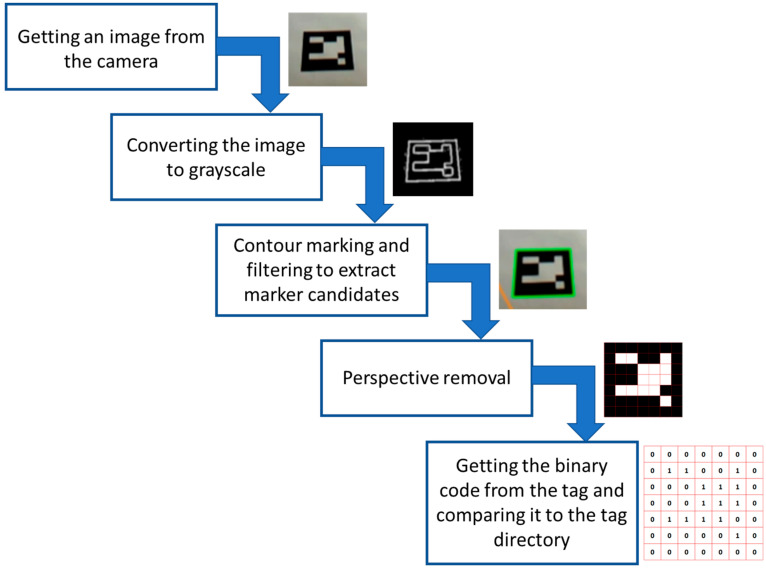
Marker detection process.

The primary purpose for which the Aruco markers were designed was to quickly determine the three-dimensional position of the camera relative to a single marker. Here, the Hamming coding algorithm was applied. The tag detection algorithm was optimized for a low false-detection rate. We could distinguish five stages of the detection process (Figure 5). After the entire algorithm is completed, the marker ID and the rotation and translation vectors are generated (to determine the position of the marker).

The Hamming encoding of the internal tag matrix provides one-bit error correction detection on each binary line. Unique tag identifiers are included in the directory, which can be placed in the ArUco module or created by the user. Once the tag ID is detected, the solvePnP (Perspective-n-Point) function is used for each corner of the tag. This function returns a list of all the possible solutions (a solution is a <rotation vector, translation vector> couple). Then, after solving the equation (Equation (1)), the 3D location of the point in is determined based on the 2D image.

(1) $$s\left[\begin{array}{c} u\\ v\\ 1 \end{array}\right]=\left(\begin{array}{ccc} f_{x} & \gamma & c_{x}\\ 0 & f_{y} & c_{y}\\ 0 & 0 & 1 \end{array}\right)\left[\begin{array}{ccc} r_{11} & r_{12} & r_{13}\\ r_{21} & r_{22} & r_{23}\\ r_{31} & r_{32} & r_{33} \end{array}\;\;\begin{array}{c} t_{1}\\ t_{2}\\ t_{3} \end{array}\right]\left[\begin{array}{c} X_{G}\\ Y_{G}\\ Z_{G}\\ 1 \end{array}\right]$$

where the vector $\left[\begin{array}{c} u\\ v\\ 1 \end{array}\right]$ describes the position of the point on the image *(u, v*); $\left(\begin{array}{ccc} f_{x} & \gamma & c_{x}\\ 0 & f_{y} & c_{y}\\ 0 & 0 & 1 \end{array}\right)$ and $\left[\begin{array}{ccc} r_{11} & r_{12} & r_{13}\\ r_{21} & r_{22} & r_{23}\\ r_{31} & r_{32} & r_{33} \end{array}\;\begin{array}{c} t_{1}\\ t_{2}\\ t_{3} \end{array}\right]$ stand for the optics parameters; and *X_G_*, *Y_G_*, and *Z_G_* describe the point in space based on the camera reference system. After placing the plane over the four points, the algorithm determines rotation vectors and translation between the camera and marker planes.

### 3.3. Automatic Control Algorithm

**Figure 6:**
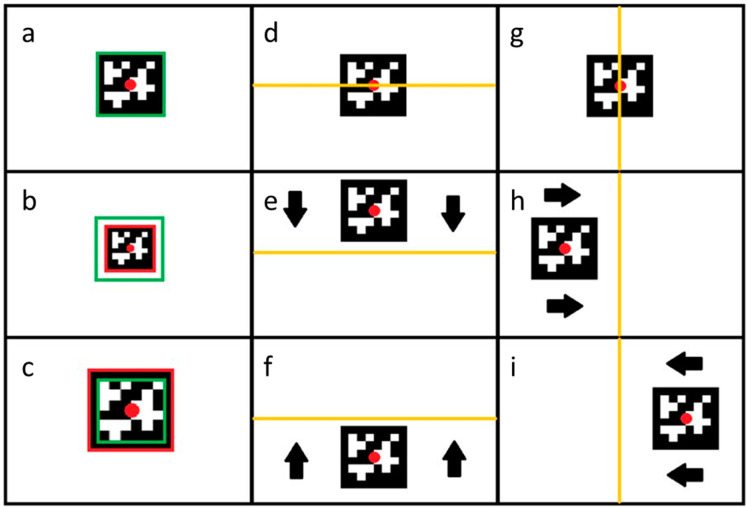
All conditions of the marker position. (**a**) desired distance—no action; (**b**) too far—move forward; (**c**) too close—move backward; (**d**) desired vertical position; (**e**) too low—move up; (**f**) too high—move down; (**g**) desired horizontal position; (**h**) too right—move left; (**i**) too left—move right.

The “leader” drone is tracked automatically. The algorithm applied works continuously in real time. By detecting the marker, the drone determines its center and locates it by taking into account the center of the field of view. On this basis, it determines the direction in which it must move and the speed it should maintain to avoid losing the marker from the field of view (Figure 6).

The first column shows the drone’s behavior in the case of forward and backward movement (movement along the *X*-axis). In this case, the determining factor for the drone’s behavior is the detected marker (Figure 6a) size. If the detected marker covers a smaller area than the assumed area (Figure 6b), the drone receives the command “move forward”, because the marker is too far away. Case (Figure 6c) describes a situation wherein the detected marker is too large, which means that the distance between the “leader” drone and the tracking drone is too small. Therefore, the drone receives the command to “move backward” and changes its position to obtain the optimal position.

The drone’s behavior along the *Z*-axis is shown in the second column. The decisive factor for the command sent to the FC is the position of the indicator’s center (Figure 6d). When the center of the marker is above the center of the field of view, the drone receives information that it is too high and must decrease the flight altitude (Figure 6e), while when the marker is below the center indicated in the image, the drone receives information that it is too low and must increase the flight altitude (Figure 6f).

The situation is similar for determining the required flight trajectory according to the *Y*-axis (Figure 6g). When the center of the marker is on the left side of the image center, the drone receives the command to “move to the right” (Figure 6h), while when the marker is on the right side of the image center, it receives the command “move to the left” (Figure 6i).

A PI controller was used to eliminate overshoots. The proportional and integral coefficients were determined and applied to the following algorithm:

In the first step, the difference between the marker area and the arithmetic average of the declared marker size range (fberror) in relation to close range (*Fbc*), away range (*Fba*), the declared max and min values of the distance between the drone and the marker (*fbrange*), and the marker area (Area) was calculated (Equation (2)).

In the second step, the difference between the marker area and the arithmetic average of the declared marker size range (fberror2) in relation to *Fb* back speed (*Fbb*), *Fb* forward speed (*Fbf*), and the declared maximum speed values (*fbspeedrange*) was calculated (Equation (3)).

Finally, a speed value (*Fbspeed*) in relation to the proportional term (*P*), the integral term (I), the previous loop error (*Pfberror*), *fberror*, and *fberror2* was calculated (Equation (4)).

In addition, to avoid exceeding the speed limits for the follower drone, we decided to protect it using a conditional statement (Equation (5)).

(2) $$fberror=area-\frac{fbc+fba}{2}$$

(3) $$fberror2=\frac{\left(fberror-fbc\right)*\left(fbf-fbb\right)}{fba-fbc}+fbb$$

(4) $$fbspeed=\frac{P*fberror2+I*\left(fberror2-pfberror\right)}{100}$$

(5) $$fbspeed=\{\begin{array}{c} -2\;\;\;\;\;\;\;\;\;fbspeed<-2\\ fbspeed\;\;\;\;fbspeed\in \left[-2,\;2\right]\\ 2\;\;\;\;\;\;\;\;\;fbspeed>2 \end{array}$$

**Figure 7:**
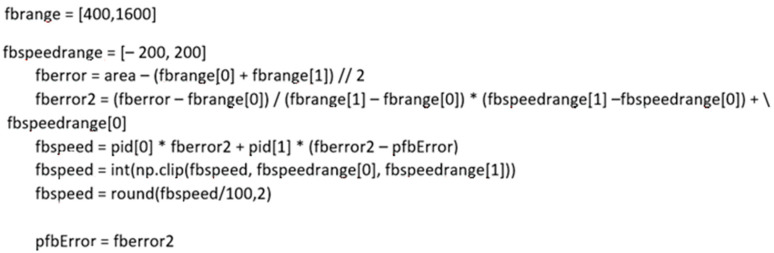
Programmatic implementation of the control algorithm.

The program code performing the controller function is shown in detail in Figure 7.

## 4. Results

**Figure 8:**
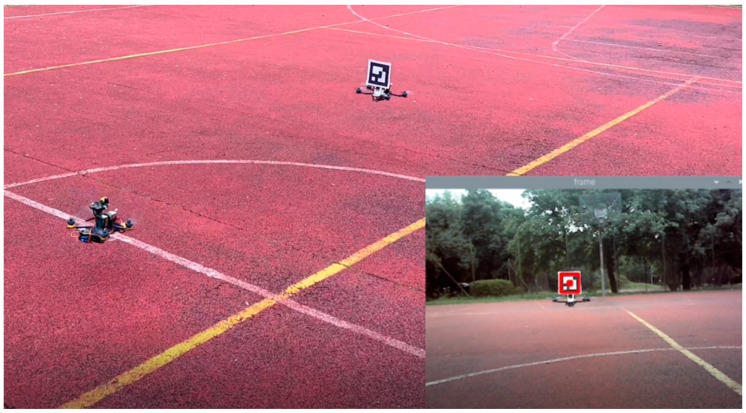
Flight in a straight line behind the leader. The bottom right image was taken by the camera, showing the detected marker.

Due to the desire to use the reconnaissance drone tracking system in unfamiliar surroundings, we did not consider the speed of following the “leader” drone. The main element of the test was to determine the forward speeds at which the flight would be smooth without losing the marker. For this purpose, the ArUco marker was installed on an IRIS 3DR UAV. The IRIS 3DR could plan the mission’s route and the speed of movement. The measurements were conducted for the three selected speeds using a 12 × 12 cm marker, which had the highest recognizability (Figure 8).

The research was divided into two parts. The first study was designed to determine the minimum corridor necessary for a safe flight. The corridor was calculated by having the drones repeatedly follow the leader moving at a constant speed in a straight line. The speeds were selected based on the limitations of the data processing steps for routing and decision making by the leader drone.

**Figure 9:**
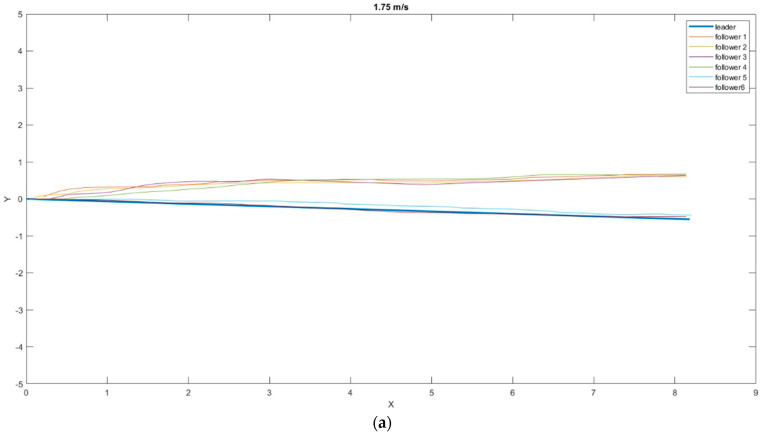

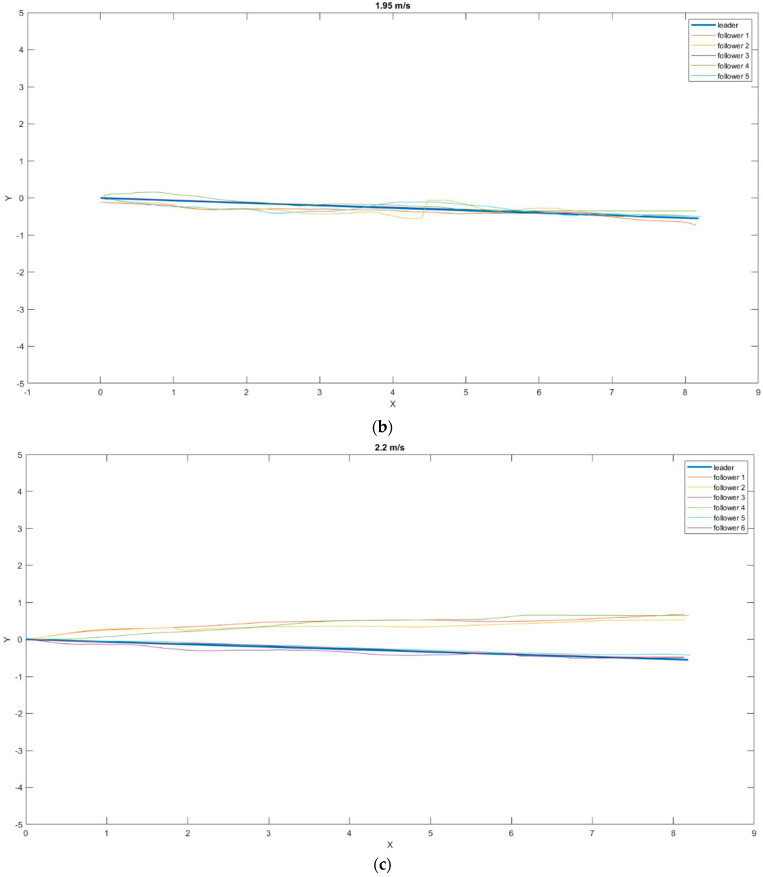
Position of the drones relative to the leader in the XY plane at different speeds: (**a**) 1.75 m/s; (**b**) 1.95 m/s; (**c**) 2.2 m/s.

The determined properties were superimposed on a single chart, while the initial position of the leader was compared to the location at which the tracking drones started the tracking process to facilitate the route analysis of the individual followers (Figure 9).

The results were obtained by subtracting each axis’s leader and follower route parameters. The data obtained this way were averaged using the arithmetic mean as the best approximation of the actual value. In contrast, to calculate the average “dispersion” of individual results around the mean value, the standard deviation from the mean was calculated with the following Equations (6)–(8).

(6) $$\bar{X}=\frac{\sum_{i=1}^{k}\left(\left|x_{Li}-x_{F_{i}}\right|\right)}{k}$$

(7) $$\bar{Y}=\frac{\sum_{i=1}^{k}\left(\left|y_{Li}-y_{F_{i}}\right|\right)}{k}$$

(8) $$\sigma =\frac{\sum_{i=1}^{k}{\left(x_{i}-\mu \right)}^{2}}{N}$$

where |$x_{Li}-x_{F_{i}}$| and $\left|y_{Li}-y_{F_{i}}\right|$ are the absolute values of the distance between the leader and the follower in a given plane.

At the specified speeds, no tracking loss of the ArUco tag was registered, and the leader’s tracking was smooth. Table 3 shows values for the mean distance to the marker on the XY plane and the standard deviations at a speed of 1.75 m/s.

Rejecting the last two measurements, which were much better than the others, and averaging the obtained results, the minimum safe corridor that would allow the drones to follow the leader had a diameter of 0.7284 m, with an SD of 0.3045 m.

In the case of measurements at 1.95 m/s, the average value of the safe corridor was lower and amounted to a surprising 0.1326 m, with an SD of 0.053 m. The distance values of the individual drones from the leader in the XY plane are presented in Table 4. Only five followers were included in this dataset, because the data from one flight were corrupted.

Comparable results to those obtained at 1.75 m/s can be seen in the third measurement at 2.2 m/s. The average safe corridor value was 0.7025 m, with an SD of 0.3044. The distance values of the individual drones from the leader in the XY plane are presented in Table 5.

**Figure 10:**
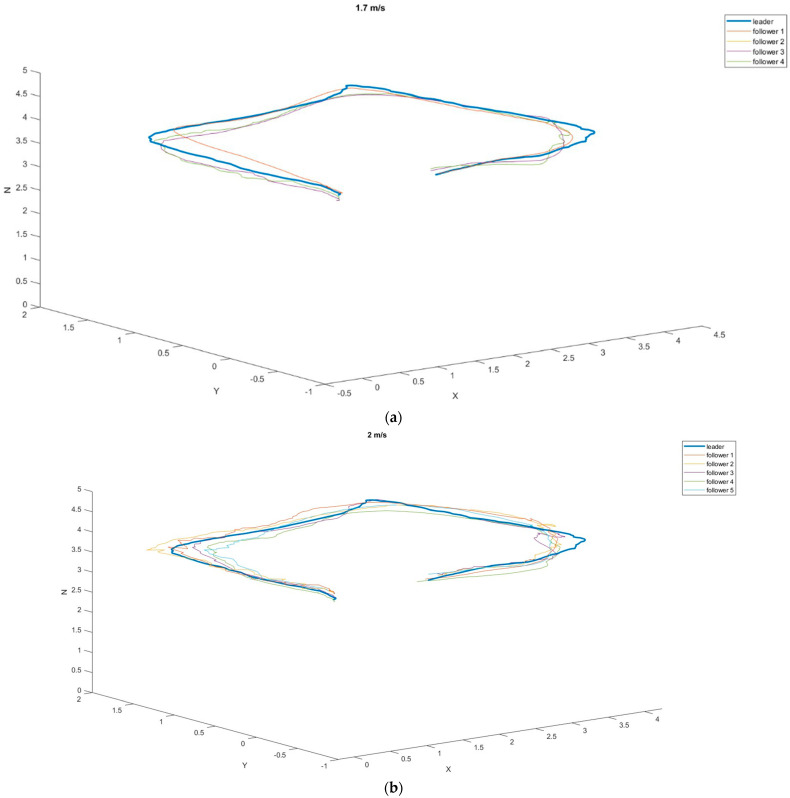

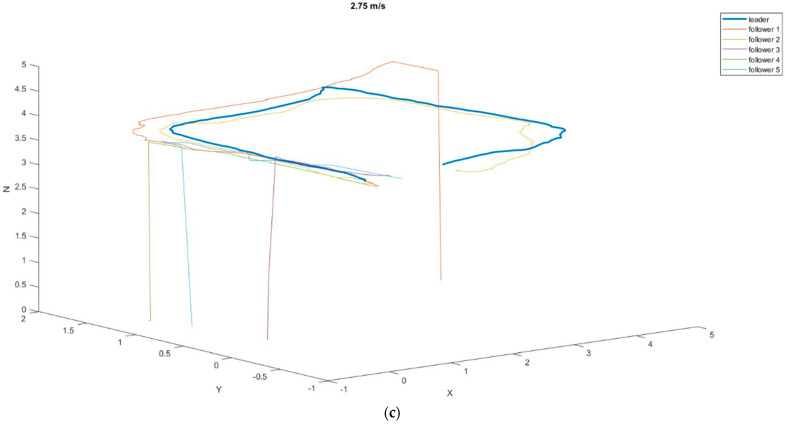
The 3D position of the drones relative to the leader at different speeds during flight around the perimeter of the rectangle: (**a**) 1.7 m/s; (**b**) 2 m/s; (**c**) 275 m/s.

In the next step, we decided to examine how the tracking drones behaved when following the leader along the programmed route. A route in the form of a rectangle with sides of 4 m and 2.5 m was chosen. The leader’s set speeds were 1.7 m/s, 2 m/s, and 2.75 m/s (Figure 10).

It can be noticed in the attached graphs that at the speeds of 1.7 m/s and 2 m/s, the tracking drones did not lose the leader over the entire route, while at the speed of 2.75 m/s, four out of five tracking attempts ended the marker being lost from sight (Table 6).

In the case of flights at a speed of 1.7 m/s, the followers’ routes were closest to the leader’s route (average distance 0.1948 m, SD 0.1114 m). Satisfactory results were also achieved at a speed of 2 m/s. According to Table 7, all followers moved at similar distances from the leader’s route. The value of the average safe corridor, in this case, was 0.2418 m, and the SD was 0.1475.

Figure 9c shows tracking blackouts for followers 1, 3, 4, and 5, which ended the mission early (for safety reasons, a landing procedure was established when the marker was lost). Due to only one drone completing the task, the mean values and standard deviations were not counted. In addition, it was recognized that the leader’s flight speed of 2.75 m/s in open space disqualified the possibility of using such speeds in missions.

## 5. Discussion

Preliminary tests determined the boundary conditions at which the system operated satisfactorily. The best recognition results for ArUco markers in terms of the distance from the camera were obtained at the marker size of 12 × 12 cm (5.86 m), while for the sizes of 6 × 6 cm and 3 × 3 cm, detection was possible at distances of 3.2 m (45% less than the best result) and 1.74 m (70% less than the best result), respectively. In addition, the best angular recognition results were also achieved for the 12 × 12 cm marker. The achieved marker deflection angles, at which the marker was still detectable, of 50.5° left and 56° right exceeded the recognition capabilities of the system for the 6 × 6 cm and 3 × 3 cm markers by 11.5% and 22.8% (46° left and 44° right) and 23.1% and 47.4% (40° left and 30° right), respectively. The results of the tests carried out under the conditions of a sunny day (63.7 klx) did not differ significantly from those of the tests carried out under shaded conditions (770 lx). The only significant differences were observed in the marker recognition distance, which increased by an average of 15% with a lower light intensity. In addition, the maximum angle of view of the camera at which marker recognition was still possible was set at 38°.

Our major research task was to determine the flight parameters at which the tracking process would be continuous and smooth while maintaining the flight trajectory as close as possible to that set by the leader. Two scenarios were provided for in the research. The first, i.e., a straight flight behind the leader at a constant speed, was used to optimize the tracking system and determine the optimal speed of the leader. Six repetitions of the flight behind the leader were carried out for the first speed adopted for the tests—1.75 m/s. The fifth and sixth attempts achieved the best results, with an average distance of 0.1868 m and 0.1341 m, respectively, from the leader’s flight trajectory on the 8 m planned route. The results from these two measurements were so favorable (78% lower than the rest of the average results obtained in this experiment) that we decided not to take them into account in the tests determining the average safe corridor that must be provided for the follower to complete the mission. Such a discrepancy in the results could have been due to the ideal weather conditions (windless day) in which these two flights were performed. The flights of the other four followers were similar to each other. The average distances from the leader’s flight trajectory were 0.7626 m, 0.7181 m, 0.7087 m, and 0.7243 m, respectively. In the case of flights at a speed of 1.95 m/s, particularly satisfactory results were obtained, with an average distance from the leader’s trajectory of 0.1326 m. The resulting distance was less than 3 cm in the consecutive tests with a leader flight speed of 2.2 m/s and 1.75 m/s.

In the second scenario, flights were carried out along a planned rectangular route. This test was performed in order to check the traceability of the tag in the proposed system. The leader’s speeds were pre-determined for the tests, just as in the first part of the study. Thus, the leader was moving at speeds of 1.7 m/s, 2 m/s, and 2.75 m/s. In the case of the first two speeds, the average distances from the leader’s trajectory were similar (0.1948 m for the speed of 1.7 m/s and 0.2418 m for 2 m/s), while the third speed turned out to be too high for the followers to keep up with the leader. Out of five attempts, only one was successful, and the follower reached the route’s endpoint with an average distance from the leader’s trajectory of 0.22 m.

According to the results of the conducted research, it can be assumed that rescue missions carried out based on the proposed system could be successful. The obtained results suggest that the organization of such tasks in an automatic system is realistic and, most importantly, effective. Of course, there are some limitations to the use of such a procedure. The main problem is the aerodynamic drag surface of the ArUco marker. Despite good tracking results, difficulties resulting from strong gusts of wind (which cannot be ruled out in open-air operations) substantially reduced the usefulness of the entire system. In closed rooms, the detection of all markers at no more than 150 cm from the “leader” UAV was achieved with 100% efficiency, while the detection of the same markers in an open space under windy weather conditions was occasionally unsuccessful, and a new procedure for finding the marker was required. In addition, it seems reasonable that a communication system should be created for the leader, connecting it to a tracking drone to avoid losing the marker. In such a case, the leader could receive information in the form of a “STOP” command, which would remain until the follower rediscovered the marker.

The great advantage of the system is that it can move freely in limited spaces where it is impossible to use GNSS navigation systems. The achieved tracking speeds corresponded to the movement of drones in an unknown space with the continuous analysis of the surrounding image. High speeds are not required in such situations, but high maneuverability is expected, which is ensured using a multi-rotor platform.

Another advantage of the proposed solution is the possibility of cascading drones depending on the amount and weight of the equipment needing to be transported. The only modification that would have to be made is that each tracking drone would have to be equipped with an ArUco marker and would become a “guide” for the next tracking drone.

The flight duration for this type of task is related to the battery used. If an extended flight time is required, a battery with a larger capacity must be used. However, it should be remembered that as the power reserve of batteries increases, their weight also increases, which is crucial considering the possibility of transporting equipment necessary to save lives and protect health.

In further studies on this project, we plan to replace the ArUco markers with infrared diodes. With such a modification, drones could track the leader even in conditions without lighting. In addition, it would eliminate problems resulting from the resistance to movement set by the marker. Another option to improve the system is to mount the camera on a gimbal placed on the drone. The proposed solution would reduce the probability of losing the tracked marker resulting from a sudden direction change by the leader.

Additionally, tests should be carried out in closed rooms, for which this system was also designed, to verify the system. In this way, we would limit the impact of external factors (such as gusts of wind, precipitation, or dust) on the entire system. Determining the characteristics of the follower and leader movement in closed rooms would contribute to creating a list of minimum requirements that must be met to use the system safely and effectively in rescue missions.

## 6. Conclusions

The proposed system could prove effective in the assumed scenarios of rescue missions. Our research found that its use in closed spaces and outdoors was possible and practical. The system had certain limitations, such as the impossibility of its use in intense winds or during missions conducted in complete darkness. The system’s capabilities could be increased, and it could be used in the dark. Using such a system would substantially reduce the cost of multiple-vehicle drone operations, but the most significant advantage of this solution is its minimization of the threat to the lives and health of rescuers who would otherwise have to perform the mission independently. Its main benefit is the innovative way of organizing the group of robots within a leader–follower formation without active communication between agents in the group or between the agent and the ground control station. This results in a considerably lower cost of expanding the group with further agents, which we identified as one of the primary drawbacks of leader–follower formations. Moreover, the system can be used immediately, without prior preparation, saving the time usually needed to perform reconnaissance and decide on how to carry out a mission.

## Figures and Tables

**Table 1:** Measurement results at 770 lx light intensity.

Marker Size [cm]	Max Distance [cm]	Max Angle L [ᵒ]	Max Angle R [ᵒ]
3 × 3	174	38.5	30
6 × 6	320	45	43
12 × 12	586	50.5	56

**Table 2:** Measurement results at 63.7 klx light intensity.

Marker Size [cm]	Max Distance [cm]	Max Angle L [ᵒ]	Max Angle R [ᵒ]
3 × 3	132	40	30
6 × 6	257	46	44
12 × 12	574	52	57

**Table 3:** The average distance and standard deviation between the follower and leader routes at a speed of 1.75 m/s.

	Follower 1	Follower 2	Follower 3	Follower 4	Follower 5	Follower 6
Arithmetic average (m)	0.7626	0.7181	0.7087	0.7243	0.1868	0.1341
Standard deviation (m)	0.2785	0.2907	0.2807	0.3680	0.0996	0.0776

**Table 4:** The average distance and standard deviation between the follower and leader routes at a speed of 1.95 m/s.

	Follower 1	Follower 2	Follower 3	Follower 4	Follower 5
Arithmetic average (m)	0.1379	0.1369	0.0499	0.1896	0.1489
Standard deviation (m)	0.039	0.0696	0.0171	0.0798	0.0549

**Table 5:** The average distance and standard deviation between the follower and leader routes at a speed of 2.2 m/s.

	Follower 1	Follower 2	Follower 3	Follower 4	Follower 5	Follower 6
Arithmetic average (m)	0.7223	0.6343	0.0837	0.7511	0.0675	0.1830
Standard deviation (m)	0.2929	0.2622	0.0452	0.3582	0.0283	0.2008

**Table 6:** The average distance and standard deviation between the follower and leader routes at a speed of 1.7 m/s during flight around the perimeter of the rectangle.

	Follower 1	Follower 2	Follower 3	Follower 4
Arithmetic average [m]	0.1655	0.1982	0.1896	0.2312
Standard deviation [m]	0.1113	0.1160	0.1126	0.0934

**Table 7:** The average distance and standard deviation between the follower and leader routes at a speed of 2 m/s in flight around the perimeter of the rectangle.

	Follower 1	Follower 2	Follower 3	Follower 4	Follower 5
Arithmetic average (m)	0.2334	0.2301	0.2276	0.2642	0.2537
Standard deviation (m)	0.1109	0.1129	0.1506	0.1827	0.1802

## Data Availability

Not applicable.
